# Eating behavior and sleep habit problems and their correlation with symptoms in children with ADHD comorbid with overweight or obesity

**DOI:** 10.1186/s13034-025-00954-w

**Published:** 2025-08-31

**Authors:** Xifei Wang, Xiaojing Yue, Lili Wang, Feiyong Jia, Honghua Li

**Affiliations:** 1 Department of Developmental and Behavioral Pediatrics, Children’s Medical center, The First Hospital of Jilin University, No. 1 Xinmin Street, Changchun, 130021 Jilin Province China; 2 The Child Health Clinical Research Center of Jilin Province, Changchun, China; 3 Department of Social Medicine and Health Management, School of Public Health, Jilin University, Changchun, Jilin Province China

**Keywords:** Eating behavior, Sleep habit, Overweight, Obesity, Attention deficit hyperactivity disorder

## Abstract

**Background:**

Previous studies have reported eating and sleep issues in children with attention deficit hyperactivity disorder (ADHD), but few have focused on those comorbid with overweight/obesity. This study aimed to investigate eating behavior and sleep habit problems in children with ADHD and comorbid overweight/obesity, and their relationship with ADHD core symptoms in such children.

**Methods:**

The study included 124 children with ADHD and overweight/obesity and 145 children with ADHD and normal weight. The Children’s Eating Behavior Questionnaire, Children’s Sleep Habit Questionnaire, and Vanderbilt ADHD Diagnostic Parent Rating Scale were used to assess eating behavior problems, sleep habit problems, and core ADHD symptoms, respectively.

**Results:**

Compared to the normal-weight children with ADHD, those with overweight/obesity scored higher in food responsiveness, enjoyment of food, night waking, sleep-disordered breathing, and daytime sleepiness, while lower in satiety responsiveness, slowness in eating, and emotional undereating. Lower scores of satiety responsiveness and slowness in eating were associated with the presence of overweight or obesity among children with ADHD. In the ADHD overweight/obesity group, shorter sleep duration was correlated with higher body mass index (BMI), and higher food responsiveness was associated with more severe attention deficit symptoms.

**Conclusions:**

Children with ADHD and overweight/obesity reported more eating and sleep problems than those with normal weight. Lower satiety response and faster eating rate may be potential risk factors for overweight/obesity in children with ADHD. Among those with comorbid overweight/obesity, shorter sleep duration associates with higher BMI, and greater food responsiveness relates to more severe attention deficit symptoms. These findings suggest eating and sleep behaviors should be considered in ADHD management, with further research needed on targeted interventions.

**Supplementary Information:**

The online version contains supplementary material available at 10.1186/s13034-025-00954-w.

## Introduction

Attention deficit hyperactivity disorder (ADHD) is a neurodevelopmental disorder that typically emerges in early childhood and is characterized by persistent symptoms of inattention, hyperactivity, and impulsivity that are disproportionate to developmental age [1]. The prevalence of ADHD among children and adolescents worldwide is 8%, with boys having a rate twice that of girls [2]. Overweight and obesity have become one of the widely recognized physical comorbidities of ADHD. The prevalence of overweight and obesity in 6-12-year-old children with ADHD is 18.8% and 13.5% respectively, with a trend of increasing rates with age [3]. This rate is significantly higher than that of the general population, by almost 4–6 times [4]. Children with ADHD comorbid with overweight or obesity are more susceptible to weight-related health issues, such as diabetes, hypertension, and cardiovascular diseases [5]. Additionally, they may experience an increased likelihood of psychological and behavioral problems, such as lower self-esteem, anxiety, and depression, as well as adverse changes in sleep patterns, dietary habits, and overall lifestyle [5].

One of the potential risks associated with comorbid overweight or obesity in children with ADHD is the development of poor eating behaviors and sleep habits. Eating behavior refers to a series of behavioral patterns that individuals exhibit in the processes of selecting, consuming, and terminating food. Sleep habits refer to an individual’s behaviors and habits in relation to sleep. Good eating behaviors and sleep habits are fundamental for promoting children’s growth, development, and cognitive function. Previous studies have indicated that children with ADHD are more prone to eating problems such as emotional eating, high food responsiveness, and picky eating behaviors [6, 7]. They are also more likely to have sleep problems such as difficulty falling asleep, sleep onset delay, nocturnal awakenings, early morning awakenings, sleep-disordered breathing, daytime sleepiness, and increased limb movements during sleep [8, 9]. Nevertheless, limited research has reported on the eating behavior and sleep habit problems of children with ADHD comorbid with overweight or obesity. Among the studies related to eating problems, only one cross-sectional study identified a negative correlation between body mass index (BMI), fat mass percentage (FM%) and slowness in eating in children with ADHD [10]. As for research on the sleep issues of children with ADHD and comorbid overweight or obesity, there is currently no available literature. Only one randomized controlled study conducted in adults suggested a positive correlation between circadian rhythm disruption and BMI in ADHD patients [11]. However, none of the above studies have investigated the dietary and sleep status of children with ADHD comorbid with overweight or obesity in comparison to those with normal weight.

Three potential mechanisms may explain why children with ADHD comorbid with overweight or obesity face a higher prevalence of eating and sleep-related problems. Firstly, long-term high-saturated-fat diets and obesity may hinder dopamine neurotransmission by repeatedly stimulating dopamine receptors, upregulating inflammatory and hormonal resistance pathways, potentially leading to a greater propensity for excessive eating and immediate gratification behaviors to enhance dopaminergic signals [12]. Secondly, children with ADHD and comorbid overweight or obesity have more severe executive function impairments, which may present greater challenges for controlling their diet and regulating their sleep [13, 14]. Thirdly, these children are more likely to experience unhealthy eating environments and concurrent psychological issues (low self-esteem, depression, anxiety, etc.), which could exacerbate eating and sleep problems [14]. Therefore, it is speculated that children with ADHD and comorbid overweight or obesity may show more eating behavior and sleep habit problems compared to normal-weight children with ADHD.

Most studies on the correlation between eating behavior and sleep problems and ADHD symptoms have been conducted in preschool and typically developing children. For instance, existing studies have reported that scores of food responsiveness and emotional overeating are positively correlated with both inattention and hyperactivity scores, while slowness in eating, desire to drink, and enjoyment of food linked to inattention scores, and desire to drink, slowness in eating, satiety responsiveness, and fussiness related to hyperactivity scores in preschool and school age children [15–17]. Poor sleep problems in typically developing children were linked to increased ADHD trait behaviors [18]. Limited studies on children with ADHD have shown that food responsiveness is associated with higher inattention scores, while sleep problems, including inadequate sleep, sleep-wake transition disorders, and excessive daytime somnolence are correlated with poorer attention and increased hyperactivity-impulse behaviors [10, 18, 19]. However, whether these problematic eating behaviors and sleep habits are associated with ADHD symptoms in children with comorbid overweight or obesity has received little attention in the current literature.

Considering that comorbid psychiatric symptoms such as oppositional defiant disorder, conduct disorder, anxiety, and depression have been shown to significantly influence both eating behaviors and sleep habits [20, 21], it is important to control for these factors in the statistical analyses to reduce potential confounding. In addition, physical activity is another critical factor, as reduced exercise levels may contribute to overweight or obesity and impact dietary and sleep habits in children with ADHD [22]. Therefore, both comorbid psychiatric symptoms and daily exercise time will be included as covariates in the present study’s analyses.

Given the limitations of existing research and potential confounding factors, the present study aims to explore the eating behavior and sleep habit problems of children with ADHD and comorbid overweight or obesity, and their associations with ADHD core symptoms, which may provide preliminary insights for clinical practice. We hypothesize that children with ADHD and comorbid overweight or obesity have more severe eating behavior and sleep habit problems compared to children with ADHD of normal weight, and these issues are related to their core ADHD symptoms.

## Methods

### Participants and procedures

From December 2022 to August 2023, 124 overweight/obesity children with ADHD (99 boys, 25 girls, with an average age of 8.6 ± 1.5 years) were recruited through the Department of Developmental and Behavioral Pediatrics Clinic of the Children’s medical center at the First Hospital of Jilin University. Normal-weight children with ADHD (145 individuals, 111 boys, 34 girls, with an average age of 8.5 ± 1.4 years) were recruited as case controls matched on age, sex, and sociodemographic factors (parents’ education level, residence, monthly household income) during the same period and at the same clinic. All children were of Chinese ethnicity.

ADHD was diagnosed according to the criteria outlined in the Diagnostic and Statistical Manual of Mental Disorders, Fifth Edition [1]. The Vanderbilt ADHD Diagnostic Rating Scale (including the parent rating scale and teacher rating scale) was used to assess the children’s behavior over the past six months. Parents completed the parent rating scale on the day of the visit, and teachers completed the teacher rating scale within one week. Subsequently, the children and their guardians returned to the hospital for a final diagnosis by the same senior expert. A diagnosis of ADHD was confirmed when both the parent and teacher rating scales met the criteria for either the attention deficit or hyperactivity/impulsivity dimension, with at least 6 items endorsed in at least one dimension.

Exclusion criteria for both groups: (1) Children classified as underweight according to the China’s “Screening Criteria for School-age Children and Adolescents malnutrition”(WS/T456-2014) [23]; (2) Children currently receiving medication or psychotherapy; (3) Children previous diagnosed with neuropathic disease and other neuropsychiatric disorders (e.g., epilepsy, autism spectrum disorder, intellectual disability); (4) Obesity due to organic diseases or medication (e.g., hypothyroidism, Cushing’s syndrome); (5) ADHD symptoms caused by organic diseases or medications (e.g., hyperthyroidism, epilepsy). Parents and guardians participated in this study after signing the informed consent forms. The Research Ethics Committee of the First Hospital of Jilin University (Changchun, China) approved the study protocol (2017-314).

## Measures

### Assessment of ADHD core symptoms and positive psychiatric comorbidities

The Vanderbilt ADHD Diagnostic Parent Rating Scale (VADPRS) was used for the assessment of ADHD core symptoms, which has been validated to have good reliability and validity [24–26]. The scale includes two parts: behavior and performance assessments, and it is divided into five domains: attention deficit (items 1–9), hyperactivity/impulsivity (items 10–18), oppositional defiance (items 19–26), conduct disorder (items 27–40), and anxiety/depression (items 41–47). Diagnostic thresholds require ≥ 4 positive oppositional defiant items for oppositional defiant disorder, ≥ 3 positive conduct disorder items for conduct disorder diagnosis, and ≥ 3 positive anxiety/depression items for anxiety/depression diagnosis according to scale criteria. In this study, the Cronbach’s alpha coefficient for the VADPRS was 0.739.

### Anthropometric measures

The height (in meters) and weight (in kilograms) of children were measured using a height ruler and weight scale. During the weight measurement, children were required to remove their shoes and heavy clothing. The BMI was calculated using the formula: BMI = Weight (kg)/[Height (m)]^2. The criteria for defining obesity or overweight was based on the Chinese standard “Screening for obesity and overweight among school-age children and adolescents“(standard number WS/T586–2018), which takes into account the BMI levels of children at different ages, such as 6 years old (overweight: boys ≥ 16.4, girls ≥ 16.2, obesity: boys ≥ 17.7, girls ≥ 17.5), 7 years old (overweight: boys ≥ 17, girls ≥ 16.8, obesity: boys ≥ 18.7, girls ≥ 18.5), 8 years old (overweight: boys ≥ 17.8, girls ≥ 17.6, obesity: boys ≥ 19.7, girls ≥ 19.4), 9 years old (overweight: boys ≥ 18.5, girls ≥ 18.5, obesity: boys ≥ 20.8, girls ≥ 20.4), 10 years old (overweight: boys ≥ 19.2, girls ≥ 19.5, obesity: boys ≥ 21.9, girls ≥ 21.5), 11 years old (overweight: boys ≥ 19.9, girls ≥ 20.5, obesity: boys ≥ 23.0, girls ≥ 22.7), 12 years old (overweight: boys ≥ 20.7, girls ≥ 21.5, obesity: boys ≥ 24.1, girls ≥ 23.9) [27, 28].

### Assessment of eating behavior problems

The Children’s eating behavior questionnaire (CEBQ) was developed by Jane Wardle for the assessment of eating behavior problems. It has been widely used with children aged 2–13 years in many countries. The Chinese version was used in this study and had been demonstrated good reliability and validity [29]. The questionnaire consists of 35 items, divided into 8 subscales. The first four subscales are satiety responsiveness, slowness in eating, fussiness, emotional undereating, belong to the food avoidance dimension. The last four subscales are food responsiveness, enjoyment of food, desire to drink, and emotional overeating, belong to the food approach dimension. Each item is scored using a Likert five-point scale: “never”, “occasionally”, “sometimes”, “often”, and “always”. Among them, Q3, Q4, Q10, Q16, and Q32 use reverse scoring. The total score range of the questionnaire is 34–170. In general, higher scores reflect more extreme eating behaviors. In this study, the Cronbach’s alpha coefficient of the CEBQ was 0.797.

### Assessment of sleep habit problems

The Children’s Sleep Habit Questionnaire (CSHQ) was used to assess sleep habit problems, developed by Owens and widely applied to children aged 4–12 years [30]. The Chinese version was developed by Li et al. which has good reliability and validity in Chinese children [31]. It consists of 33 items covering 8 domains: bedtime resistance, sleep onset delay, sleep duration, sleep anxiety, night waking, parasomnias, sleep-disordered breathing, and daytime sleepiness. Each item is scored using a Likert 3-point scale: rarely or none (0–1 times per week), sometimes (2–4 times per week), and often (5–7 times per week). The total score range of the questionnaire is 0–99, with a total score of ≥ 41 indicating the presence of sleep-related problems. The higher score suggests more severe sleep issues. In this study, the Cronbach’s alpha coefficient of the CSHQ was 0.858.

### Statistical analysis

Data were analyzed using IBM SPSS 26.0 statistical software. Shapiro-Wilk tests were conducted on continuous variables to assess normal distribution. Normally distributed data were presented as mean ± standard deviation (Mean ± SD); non-normally distributed data as medians ± interquartile ranges (M (P25-P75)); Categorical variables as percentages. Chi-square tests were used to evaluate differences in unordered categorical variables between the two groups. Independent samples t-tests were employed to assess between-group differences for normally distributed continuous variables, while the Mann-Whitney U tests were utilized for non-normal distributed continuous variables. Binary logistic regression analysis was used to analyses whether eating behavior problems and sleep habit problems were associated with co-morbid overweight obesity in children with ADHD. Pearson’s correlation analysis was applied to assess the correlation between ADHD symptoms and eating/sleep problems, as well as between BMI and sleep problems. Spearman’s correlation analysis was applied to assess the correlation between positive psychiatric comorbidities and eating/sleep problems. Multivariate linear regression analysis was performed on independent variables that were statistically significant in univariate analysis. The significance level was set at a two-tailed *P*-value of < 0.05.

## Results

### Demographics of the ADHD normal weight group and ADHD overweight/obesity group

Table 1 presents the demographic statistics for both groups. The BMI of the ADHD overweight/obesity group was significantly higher compared to the normal weight group (*P* < 0.001). No significant differences were found between the ADHD normal weight group and the ADHD overweight/obesity group in terms of sex, age, ADHD subtype, birth weight, gestational age, parents’ education level, parents’ marital status, residence, monthly household income, daily screen exposure time or daily exercise time (*P* > 0.05).

**Table 1:** Demographic characteristics of the ADHD normal weight group and ADHD overweight/obesity group

Variable		ADHD normal weight group (*n* = 145)	ADHD overweight/obesity group (*n* = 124)	t/X^2^	*P*
BMI (kg/m^2^)		15.4 ± 1.8	23.4 ± 5.2	−16.303	**< 0.001**
Sex	Male (%)	111 (76.6%)	99 (79.8%)	0.422	0.516
Age (years)		8.5 ± 1.4	8.6 ± 1.5	−0.611	0.541
ADHD subtypes	Inattention dominant type	95 (65.5%)	75 (60.5%)	4.058	0.131
	Hyperactivity/Impulse dominant type	16 (11.0%)	8 (6.5%)		
	Combined type	34 (23.4%)	41 (33.1%)		
Birth weight (kg)		3.4 ± 0.6	3.4 ± 0.6	0.145	0.885
Birth weight	Low birth weight	7 (4.8%)	5 (4.0%)	0.143	0.931
	Normal birth weight	117 (80.7%)	102 (82.3%)		
	Macrosomia	21 (14.5%)	17 (13.7%)		
Gestational age (week)		39.1 ± 2.0	39.0 ± 2.2	0.664	0.507
Gestational age	Preterm	12 (8.3%)	10 (8.1%)	0.448	0.799
	Term	126 (86.9%)	110 (88.7%)		
	Post-term	7 (4.8%)	4 (3.2%)		
Father’s education level	Primary school and below	7 (4.8%)	9 (7.3%)	0.749	0.688
	Junior/Senior School	75 (51.7%)	64 (51.6%)		
	Bachelor’s degree and above	63 (43.4%)	51 (41.1%)		
Mother’s education level	Primary school and below	7 (4.8%)	6 (4.8%)	0.688	0.709
	Junior/Senior School	63 (43.4%)	60 (48.4%)		
	Bachelor’s degree and above	75 (51.7%)	58 (46.8%)		
Parents’ marital status	Married	131 (90.3%)	104 (83.9%)	2.537	0.111
	Divorced/widowed/single	14 (9.7%)	20 (16.1%)		
Residence	Urban area	121 (83.4%)	100 (80.6%)	0.358	0.549
	Rural area	24 (16.6%)	24 (19.4%)		
Monthly household income (¥)	≤ 2000	5 (4.1%)	7 (6.9%)	4.908	0.297
	2000–5000	42 (34.7%)	28 (27.5%)		
	5000–10,000	45 (37.2%)	46 (45.1%)		
	10,000–20,000	21 (17.4%)	11 (10.8%)		
	≥ 20,000	8 (6.6%)	10 (9.8%)		
Daily screen exposure time (hour)	< 1	82 (58.6%)	75 (62.0%)	0.315	0.574
	≥ 1	58 (41.4%)	46 (38.0%)		
Daily exercise time (hour)	< 1	84 (59.2%)	69 (57.5%)	0.073	0.787
	≥ 1	58 (40.8%)	51 (42.5%)		

### Comparation of the eating behavior and sleep habit problems between the ADHD normal weight group and ADHD overweight/obesity group

Table 2 exhibits the CEBQ and the CSHQ scores of two groups. In terms of eating behavior problems, compared with ADHD normal weight group, ADHD overweight/obesity group exhibited lower scores in satiety responsiveness, slowness in eating and emotional undereating, whereas higher scores in food responsiveness and enjoyment of food (*P* < 0.05). No statistically significant differences were observed in the scores of fussiness, desire to drink, and emotional overeating between two groups (*P* > 0.05). In terms of sleep habit problems, the ADHD overweight/obesity group showed significantly higher scores in night waking, sleep disordered breathing, daytime sleepiness, and total score of CSHQ (*P* < 0.05). No significant differences were observed between the two groups in the scores for bedtime resistance, sleep onset delay, sleep duration, sleep anxiety, and parasomnias (*P* > 0.05).

**Table 2:** Eating behavior and sleep habit problems of the ADHD normal weight group and ADHD overweight/obesity group

Variable		ADHD normal weight group (*n* = 145)	ADHD overweight/obesity group (*n* = 124)	t	*P*
Eating behavior problems	Satiety responsiveness	10.4 ± 4.4	6.6 ± 3.8	7.650	**< 0.001**
	Slowness in eating	8.4 ± 4.3	5.1 ± 4.0	6.451	**< 0.001**
	Fussiness	10.6 ± 5.1	10.6 ± 4.5	−0.078	0.938
	Emotional undereating	5.9 ± 4.1	4.9 ± 3.8	2.069	**0.040**
	Food responsiveness	4.3 ± 3.7	5.9 ± 4.5	−3.057	**0.002**
	Enjoyment of food	7.7 ± 4.3	10.2 ± 4.6	−4.153	**< 0.001**
	Desire to drink	5.0 ± 3.4	5.0 ± 3.5	0.030	0.976
	Emotional overeating	1.8 ± 2.4	1.9 ± 3.2	−0.256	0.798
Sleep habit problems	Bedtime resistance	11.8 ± 1.9	11.7 ± 1.9	0.278	0.781
	Sleep onset delay	2.6 ± 0.6	2.5 ± 0.7	0.955	0.335
	Sleep duration	6.4 ± 1.4	6.5 ± 1.4	−0.344	0.731
	Sleep anxiety	6.9 ± 2.1	7.3 ± 2.2	−1.409	0.160
	Night waking	3.7 ± 1.3	4.1 ± 1.8	−2.460	**0.015**
	Parasomnias	8.6 ± 2.4	9.2 ± 3.4	−1.497	0.136
	Sleep disordered breathing	3.5 ± 1.2	3.9 ± 1.6	−2.314	**0.022**
	Daytime sleepiness	9.6 ± 2.6	10.3 ± 2.8	−2.160	**0.032**
	Total sleep habit problems score	49.0 ± 7.2	51.3 ± 9.8	−2.190	**0.030**

### Comparation of the ADHD core symptoms and comorbid psychiatric symptoms between the ADHD normal weight group and ADHD overweight/obesity group

Table S1a exhibits the scores of attention deficit and hyperactivity/impulsivity, and psychiatric comorbidity status of two groups, revealing no significant differences (*P* > 0.05). Table S1b details the positive item counts for psychiatric comorbidities, with no statistically significant between-group differences observed.

### Associations of eating behavior and sleep habit problems on the presence or absence of overweight or obesity in children with ADHD

In terms of eating behavior problems, binary logistic regression analyses revealed that satiety responsiveness scores (unadjusted OR = 0.856, 95% confidence interval (CI) 0.788 to 0.930; *P* < 0.001) and slowness in eating scores (unadjusted OR = 0.903, 95%CI 0.840 to 0.969; *P* = 0.005) were significantly associated with the presence of overweight or obesity in children with ADHD. After adjusting for sex, age, daily exercise time, positive item counts of oppositional defiant disorder, conduct disorder, and anxiety/depression, the associations between satiety responsiveness scores (adjusted OR = 0.846, 95%CI 0.775 to 0.923; *P* < 0.001) and slowness in eating scores (adjusted OR = 0.893, 95%CI 0.829 to 0.963; *P* = 0.003) with the presence of overweight or obesity in children with ADHD remained significant (Table 3). In terms of sleep habit problems, logistic regression analyses showed that the scores for night waking, sleep disordered breathing, and daytime sleepiness were not significantly associated with the presence of overweight or obesity in children with ADHD (Table 3).

**Table 3:** Associations of eating behavior and sleep habit problems on the presence or absence of overweight or obesity in children with ADHD

	Model 1^a^					Model 2^b^					Model 3^c^				
	B	Wald	*P*	OR	95%CI	B	Wald	*P*	OR	95%CI	B	Wald	*P*	OR	95%CI
Food responsiveness	0.073	3.680	0.055	1.075	(0.998, 1.158)	0.076	3.827	0.050	1.079	(1.000, 1.164)	0.074	3.551	0.060	1.077	(0.997, 1.163)
Enjoyment of food	0.024	0.421	0.517	1.024	(0.953, 1.101)	0.014	0.132	0.716	1.014	(0.942, 1.091)	0.016	0.191	0.662	1.017	(0.944, 1.094)
Satiety responsiveness	−0.155	13.578	**< 0.001**	0.856	(0.788, 0.930)	−0.163	13.760	**< 0.001**	0.850	(0.780, 0.926)	−0.168	14.198	**< 0.001**	0.846	(0.775, 0.923)
Slowness in eating	−0.102	7.902	**0.005**	0.903	(0.840, 0.969)	−0.113	8.891	**0.003**	0.893	(0.829, 0.962)	−0.113	8.724	**0.003**	0.893	(0.829, 0.963)
Emotional undereating	−0.040	1.004	0.316	0.961	(0.889, 1.039)	−0.033	0.668	0.414	0.967	(0.894, 1.047)	−0.042	0.990	0.320	0.959	(0.883, 1.042)
Night waking	0.113	1.099	0.295	1.119	(0.907, 1.382)	0.109	0.985	0.321	1.115	(0.899, 1.384)	0.117	1.103	0.294	1.124	(0.903, 1.400)
Sleep disordered breathing	0.092	0.619	0.432	1.097	(0.872, 1.379)	0.089	0.563	0.453	1.093	(0.867, 1.377)	0.080	0.458	0.499	1.084	(0.858, 1.368)
Daytime sleepiness	0.058	1.261	0.261	1.059	(0.958, 1.171)	0.073	1.862	0.172	1.075	(0.969, 1.193)	0.080	2.111	0.146	1.083	(0.973, 1.206)

CI: confidence interval

^a^unadjusted model

^b^adjusted model: adjusted for sex, age, and daily exercise time

^c^adjusted model: adjusted for sex, age, daily exercise time, and item counts of oppositional defiant disorder, conduct disorder, and anxiety/depression

### Correlation between sleep habit problems and BMI in ADHD overweight/obesity group

In children with ADHD and comorbid overweight or obesity, correlation coefficients for sleep habit problems and BMI are shown in Table S2a. Based on the linear regression analysis, the sleep duration was significantly negatively correlated with BMI (unadjusted β = −0.195, 95%CI −1.351 to −0.070; *P* = 0.030; adjusted β = −0.214, 95%CI −1.442 to −0.112; *P* = 0.022) (Table S2b). For every one-unit decrease in the sleep duration score, BMI increased by 0.777 units.

### Correlation between eating behavior and sleep habit problems with ADHD core symptoms in ADHD overweight/obesity group

In children with ADHD and comorbid overweight or obesity, correlation coefficients between eating behavior scores and ADHD core symptom scores as well as psychiatric comorbidity scores are outlined in Table S3a. Based on the multiple linear regression analysis, the food responsiveness score was significantly correlated with the attention deficit score in both the unadjusted and adjusted models (unadjusted β = 0.287, 95%CI 0.104 to 0.413; *P* = 0.001; adjusted β = 0.312, 95%CI 0.107 to 0.462; *P* = 0.002) (Table S3b). For every one-unit increase in the food responsiveness score, the attention deficit score increased by 0.285 units.

Table S4 presents the correlation coefficients between sleep habit scores and ADHD core symptom scores as well as psychiatric comorbidity scores, no significant correlation between sleep habit scores and ADHD core symptom scores were found.

### Sensitivity analysis

We recognize that z-scores offer greater advantages for international research comparisons. Therefore, we conducted a sensitivity analysis by reclassifying children using the WHO-standard body mass index-for-age z-score (BMIZ) into: normal weight (−2 SD ≤ BMIZ < + 1 SD) and overweight/obesity (BMIZ ≥ + 1 SD) groups [32].

First, classification based on WHO BMIZ demonstrated high agreement with Chinese BMI percentile-based criteria (Kappa = 0.918) (Table S5). Additionally, univariate analyses for both groups on demographic characteristics, eating behavior problems, sleep habit problems, ADHD symptom scores, and psychiatric comorbidity scores, as well as regression analyses of eating behavior and sleep habit problems-overweight/obesity associations, all remained consistent with original findings (Tables S6–S9). Moreover, among children with ADHD and comorbid overweight/obesity defined by WHO BMIZ standard, the correlation coefficients between BMI and sleep duration (unadjusted β = −0.204, 95% CI: −1.362 to −0.127; *P* = 0.018; adjusted β = −0.213, 95% CI: −1.424 to −0.127; *P* = 0.019) and between food responsiveness and attention deficit (unadjusted β = 0.247, 95% CI: 0.072 to 0.373; *P* = 0.004; adjusted β = 0.244, 95% CI: 0.047 to 0.396; *P* = 0.013) remained consistent with the original results, indicating robustness of the current results (Tables S10–S11).

## Discussion

This study presents four major findings. Firstly, children with ADHD and comorbid overweight/obesity exhibited more eating behavior and sleep habit issues compared to normal-weight children with ADHD. They exhibited increased food responsiveness, faster eating rate, and greater enjoyment of food, and more frequent night waking, sleep-disordered breathing, and increased daytime sleepiness. At the same time, they showed reduced satiety sensitivity and lower emotional undereating. Secondly, lower satiety response and faster eating rate were associated with the presence of overweight or obesity in children with ADHD. Thirdly, in children with ADHD and comorbid overweight or obesity, sleep duration showed a negative association with BMI. Fourth, food responsiveness showed a positive association with the severity of attention deficit symptoms in children with ADHD and comorbid overweight or obesity.

Compared to normal weight children with ADHD, this study found that children with ADHD and comorbid overweight or obesity exhibit more eating behavior problems. Due to the limited research on eating behaviors in children with ADHD and comorbid overweight or obesity, comparing our findings with similar populations is difficult. In terms of food approach, Kininmonth et al.‘s meta-analysis in general pediatric populations has shown a positive link between food responsiveness, enjoyment of food and obesity measures [33]. In research on children with ADHD, a small sample size case-control study conducted in China by Wu et al. found a positive correlation between food responsiveness scores and FM% [10]. These results are consistent with our findings of higher food responsiveness and enjoyment of food scores in children with ADHD and comorbid overweight or obesity. On the other hand, in terms of food avoidance, Kininmonth et al. have shown that satiety responsiveness and slowness in eating are negatively associated with obesity measures in the general pediatric population [33]. Wu et al. have also reported a negative correlation between slowness in eating scores and BMI/FM% in children with ADHD [10]. Our study further revealed that children with ADHD and comorbid overweight/obesity scored significantly lower in the behavior of slowness in eating, satiety responsiveness, and emotional undereating. The reason for more eating behavior problems in those with overweight/obesity may partially explained by the dopamine reward imbalance hypothesis [10, 15]. High-fat diets and diet-induced obesity can chronically stimulate dopamine receptors and inhibit dopamine signaling, while promoting inflammatory processes that may impair specific cognitive functions in the cerebral cortex and hippocampus, which could contribute to dysregulation of the reward system. This dysregulation is thought to manifest as an enhanced pleasure response and diminished sensitivity to rewarding foods, along with impaired impulse control and resistance to appetite-regulating hormones, such as leptin and insulin. Consequently, children with ADHD and comorbid overweight or obesity may exhibit stronger enjoyment of food, heightened food responsiveness, faster eating speed, reduced sensitivity to satiety signals, and greater vulnerability to emotional influences on food eating behaviors [12, 34]. However, it is important to recognize that this proposed mechanism remains hypothetical and likely interacts with multiple other environmental and psychosocial factors.

To our knowledge, this represents the first case-control study to report that children with ADHD and comorbid overweight or obesity exhibit more severe sleep habit problems, including night waking, sleep-disordered breathing, and daytime sleepiness, compared to children with ADHD of normal weight. This finding is consistent with the observed association between higher weight status and increased night waking frequency in the general pediatric population, as reported by parents and measured by accelerometry [35]. Similarly, it also aligns with the reported link between obesity and higher risk of sleep-disordered breathing in the same population, as measured by polysomnography [36]. Obesity may potentially exacerbate the respiratory burden in children with ADHD through adenoid hypertrophy/hyperplasia and fat deposition, increasing the likelihood of nocturnal respiratory disruptions and nighttime awakenings, which may contribute to daytime fatigue and somnolence [37]. Additionally, alterations in the reward system in obese children may promote the consumption of low-quality foods (e.g., more processed, higher glycemic index, higher-fat content foods), which have been reported to correlate with disrupted sleep-wake cycles [38]. Weight stigma may also contribute to reduced sleep quality and duration [39]. Although previous studies among primary school-aged children and adolescents have reported an association between sleep duration and obesity [35, 40], our study in children with ADHD found no significant difference in sleep duration scores between those with overweight/obesity versus normal-weight peers. This divergence may reflect differences in the study populations, and the subjective or objective methods used for sleep reporting.

In our study, after controlling for daily exercise time, sex, age and psychiatric comorbidity item counts, regression analysis showed that lower satiety responsiveness and faster eating rate were significantly associated with the presence of overweight or obesity in children with ADHD. This suggests that satiety responsiveness and eating speed represent potential modifiable factors for weight management in children with ADHD. This finding is in line with previously studies in primary school children that reported a negative correlation between satiety responsiveness/slower eating and body weight [41, 42]. To date, no similar findings have previously been reported in children with ADHD. Slower eating has been shown to promote the secretion of anorexigenic gut peptides and facilitate earlier satiety [43]. Earlier satiety may contribute to reduced overweight/obesity risk by suppressing appetite and improving food intake regulation. Modulating children’s satiety response and eating speed through increased meal frequency, reduced portion sizes, and improved dietary quality (e.g., higher protein intake, more fruits and vegetables, and reduced fast food and snacks) could represent potential weight management strategies for children with ADHD [44, 45].

Additionally, our study found that in children with ADHD and comorbid overweight or obesity, sleep duration was negatively correlated with BMI. For every one-unit decrease in sleep duration score, BMI increased by 0.777, which indicates that shorter sleep duration may be a potential risk factor for increased BMI in this population. This association remained significant held after adjusting for confounding factors. This finding is consistent with previous studies in primary school children, which widely reported a negative association between sleep duration and weight status [35]. Shortened sleep duration may disrupt the balance of appetite-regulating hormones, leading to decreased leptin levels and increased ghrelin levels, which could exacerbate hunger and problematic eating behaviors [46]. Furthermore, inadequate sleep may increase fatigue and affect individual emotions, possibly reducing daily physical activity and extending screen time, both of which are associated with weight gain [38]. Therefore, dual interventions from both of family and school, particularly focusing on sleep duration management, such as advancing bedtime, minimizing screen exposure before sleep, and controlling nighttime eating, may mitigate the risk of weight gain in children with ADHD and comorbid overweight or obesity.

Our study also found a positive correlation between food responsiveness and attention deficit scores in children with ADHD and comorbid overweight or obesity. This finding remained significant even after controlling for potential confounding factors. Food responsiveness is defined as the impulsive reactions to external food cues. This suggests that heightened reactivity to food cues may contribute to more severe attention deficit symptoms in these children. In both preschool children and children diagnosed with ADHD, food responsiveness has been previously shown to be associated with symptoms of inattention, hyperactivity, and impulsivity symptoms [10, 15, 17]. However, our study did not find a correlation between food responsiveness and hyperactivity-impulsivity symptoms in children with ADHD and comorbid overweight/obesity. Additionally, although prior research in both the general population and children with ADHD has shown an association between ADHD core symptoms and sleep problems, our study did not find such a relationship in children with ADHD and comorbid overweight or obesity [18, 19]. The discrepancy may reflect unique study population (children with ADHD and comorbid overweight/obesity) and age-related differences. Multicenter, larger-scale studies are essential to further investigate these associations.

Although this study is the first case-control investigation to explore eating and sleep problems in children with ADHD and comorbid overweight or obesity, and has controlled for potential confounding factors, we acknowledge that there are still some limitations. First, due to the cross-sectional nature of the study, it cannot establish causality between eating or sleep problems and overweight/obesity in children with ADHD. Future longitudinal studies are needed to clarify the directionality and underlying mechanisms of these associations. Second, the assessment questionnaires relied on parent reports, which may introduce subjective bias. Incorporating objective assessments such as actigraphy for sleep and real-time dietary monitoring in future studies would improve measurement accuracy. Third, the absence of a typically developing control group without any psychiatric diagnoses limits the ability to disentangle ADHD-specific effects on BMI, eating behavior and sleep habit problems. Fourth, the sex imbalance in the ADHD with comorbid overweight/obesity group (99 boys, 25 girls) limited sex-stratified analyses, which may overlook potential gender differences in these domains. Fifth, although psychiatric comorbidities were controlled for using VADPRS, the study did not include comprehensive clinical diagnostic interviews to fully characterize the range and severity of comorbid conditions, which might affect study findings. Finally, this study did not systematically assess children’s exposure to traumatic events or maternal trauma, nor did it account for other potential confounding factors such as family environment, parenting practices, and broader social determinants. These factors may potentially influence children’s eating and sleep problems. Future research incorporating objective measures, multi-source data, and additional control variables will help to improve the objectivity and reliability of the findings.

## Conclusions

Children with ADHD and comorbid overweight or obesity show more dietary and sleep problems compared to those with normal weight. Lower satiety response and faster eating rate are associated with overweight/obesity in children with ADHD. Among children with ADHD and comorbid overweight or obesity, shorter sleep duration is associated with higher BMI, and higher food responsiveness relates to more severe attention deficit symptoms. These findings underscore the potential value of considering dietary and sleep behaviors in the clinical management of children with ADHD. Further research is needed to determine whether targeted nutritional guidance and sleep interventions could effectively support health promotion in this population.

## Supplementary Information

Supplementary Material 1

## Data Availability

The datasets used during the current study are available from the corresponding author on reasonable request.

## Abbreviations

ADHD: Attention deficit hyperactivity disorder
BMI: Body mass index
FM%: Fat mass percentage
VADPRS: Vanderbilt ADHD Diagnostic Parent Rating Scale
CEBQ: Children’s Eating Behavior questionnaire
CSHQ: Children’s Sleep Habit Questionnaire
SD: Standard deviation
CI: Confidence interval
BMIZ: Body mass index-for-age z-score
