# Supplementary material for: Eating behavior and sleep habit problems and their correlation with symptoms in children with ADHD comorbid with overweight or obesity
Source: Child Adolesc Psychiatry Ment Health. 2025 Aug 31;19:99. doi: 10.1186/s13034-025-00954-w (PMC12400763; doi:10.1186/s13034-025-00954-w)
Supplement: Supplementary file 1 — Supplementary Material 1 [file 13034_2025_954_MOESM1_ESM.docx]

**Appendices：**

**Table S1a Scores of attention deficit and hyperactivity/impulsivity, and psychiatric comorbidity status of the ADHD normal weight group and ADHD overweight/obesity group.**

|  | | ADHD normal weight group (n=145) | ADHD overweight/obesity group (n=124) | t/X^2^ | *P* |
| --- | --- | --- | --- | --- | --- |
| Attention deficit | | 19.7 ± 4.2 | 19.8 ± 4.1 | -0.256 | 0.798 |
| Hyperactivity/impulsivity | | 14.4 ± 6.2 | 14.8 ± 6.2 | -0.590 | 0.556 |
| Oppositional defiance disorder | Absent | 88 (60.7%) | 76 (61.3%) | 0.010 | 0.920 |
|  | Present | 57 (39.3%) | 48 (38.7%) |  |  |
| Conduct disorder | Absent | 140 (96.6%) | 117 (94.4%) | 0.757 | 0.555 |
|  | Present | 5 (3.4%) | 7 (5.6%) |  |  |
| Anxiety/depression | Absent | 117 (80.7%) | 101 (81.5%) | 0.025 | 0.874 |
|  | Present | 28 (19.3%) | 23 (18.5%) |  |  |

**Table S1b** **Positive** **psychiatric comorbidity** **item counts of the ADHD normal weight group and ADHD overweight/obesity group.**

|  | ADHD normal weight group (n=145) | ADHD overweight/obesity group (n=124) | z | *P* |
| --- | --- | --- | --- | --- |
| Oppositional defiance disorder | 2.0 (0.5-5.0) | 3.0 (1.0-5.0) | -0.433 | 0.665 |
| Conduct disorder | 0.0 (0.0-1.0) | 0.0 (0.0-1.0) | -0.421 | 0.674 |
| Anxiety/depression | 1.0 (0.0-2.0) | 0.0 (0.0-2.0) | -0.807 | 0.420 |

**Table S2a Correlation between sleep habits and BMI in** **children with ADHD and comorbid overweight or obesity.**

|  | 1 | 2 | 3 | 4 | 5 | 6 | 7 | 8 | 9 |
| --- | --- | --- | --- | --- | --- | --- | --- | --- | --- |
| 1 BMI | - |  |  |  |  |  |  |  |  |
| 2 Bedtime resistance | 0.056 | - |  |  |  |  |  |  |  |
| 3 Sleep onset delay | -0.043 | 0.137 | - |  |  |  |  |  |  |
| 4 Sleep duration | **-0.195*** | 0.201* | -0.028 | - |  |  |  |  |  |
| 5 Sleep anxiety | -0.029 | 0.598* | -0.230* | 0.097 | - |  |  |  |  |
| 6 Night waking | -0.028 | 0.277* | 0.076 | 0.175 | 0.243* | - |  |  |  |
| 7 Parasomnias | -0.023 | 0.300* | 0.002 | 0.209* | 0.357* | 0.742* | - |  |  |
| 8 Sleep disordered breathing | -0.039 | 0.205* | -0.053 | 0.046 | 0.301* | 0.656* | 0.805* | - |  |
| 9 Daytime sleepiness | -0.065 | 0.059 | -0.114 | 0.214* | 0.228* | 0.459* | 0.505* | 0.397* | - |

* *P*<0.05, ** *P*<0.001

**Table S2b Correlation between** **sleep habits and BMI in children with ADHD and comorbid overweight or obesity.**

|  | Model1^a^ | | | | | Model2^b^ | | | | | Model3^c^ | | | | |
| --- | --- | --- | --- | --- | --- | --- | --- | --- | --- | --- | --- | --- | --- | --- | --- |
|  | B | β | t | *P* | 95%CI | B | β | t | *P* | 95%CI | B | β | t | *P* | 95%CI |
| Sleep duration | -0.711 | -0.195 | -2.196 | **0.030** | (-1.351, -0.070) | -0.771 | -0.212 | -2.363 | **0.020** | (-1.417, -0.125) | -0.777 | -0.214 | -2.316 | **0.022** | (-1.442, -0.112) |

CI: confidence interval.

^a^unadjusted model.

^b^adjusted model: adjusted for sex, ADHD subtypes, daily screen exposure time and daily exercise time.

^c^adjusted model: adjusted for sex, ADHD subtypes, daily screen exposure time, daily exercise time and positive item counts of oppositional defiant disorder, conduct disorder, and anxiety/depression.

**Table S3a Correlation of** **eating behaviors with ADHD core symptoms** **and** **psychiatric comorbidities in children with ADHD and comorbid overweight or obesity.**

|  | 1 | 2 | 3 | 4 | 5 | 6 | 7 | 8 | 9 | 10 | 11 | 12 | 13 |
| --- | --- | --- | --- | --- | --- | --- | --- | --- | --- | --- | --- | --- | --- |
| 1 Attention deficit | - |  |  |  |  |  |  |  |  |  |  |  |  |
| 2 Hyperactivity/impulsivity | 0.230^*^ | - |  |  |  |  |  |  |  |  |  |  |  |
| 3 Oppositional defiant disorder | 0.194* | 0.360** | - |  |  |  |  |  |  |  |  |  |  |
| 4 Conduct disorder | 0.225* | 0.349** | 0.519** | - |  |  |  |  |  |  |  |  |  |
| 5 Anxiety/depression | 0.049 | -0.045 | 0.260* | 0.163 | - |  |  |  |  |  |  |  |  |
| 6 Food responsiveness | **0.287^**^** | 0.119 | 0.145 | **0.239*** | -0.083 | - |  |  |  |  |  |  |  |
| 7 Enjoyment of food | 0.091 | 0.075 | 0.041 | 0.009 | 0.022 | 0.448^**^ | - |  |  |  |  |  |  |
| 8 Desire to drink | 0.175 | 0.115 | **0.191*** | 0.103 | 0.011 | 0.364^**^ | 0.408^**^ | - |  |  |  |  |  |
| 9 Emotional overeating | 0.073 | 0.044 | -0.010 | **0.199*** | -0.017 | 0.494^**^ | 0.216^*^ | 0.271^**^ | - |  |  |  |  |
| 10 Satiety responsiveness | 0.100 | -0.076 | 0.081 | 0.117 | 0.040 | -0.098 | -0.292^**^ | 0.137 | 0.010 | - |  |  |  |
| 11 Slowness in eating | 0.014 | -0.130 | -0.053 | -0.027 | 0.105 | -0.202^*^ | -0.276^**^ | -0.098 | 0.080 | 0.489^**^ | - |  |  |
| 12 Fussiness | 0.051 | -0.136 | -0.059 | -0.115 | 0.086 | -0.203^*^ | -0.202^*^ | 0.027 | -0.120 | 0.244^**^ | 0.128 | - |  |
| 13 Emotional undereating | 0.108 | 0.048 | 0.141 | **0.204*** | **0.192*** | 0.248^**^ | 0.206^*^ | 0.386^**^ | 0.392^**^ | 0.292^**^ | 0.049 | 0.018 | - |

** P*<0.05, ** *P*<0.001

**Table S3b Correlation of food responsiveness with attention deficit in children with ADHD and comorbid overweight or obesity.**

|  | Model1^a^ | | | | | Model2^b^ | | | | | Model3^c^ | | | | |
| --- | --- | --- | --- | --- | --- | --- | --- | --- | --- | --- | --- | --- | --- | --- | --- |
|  | B | β | t | *P* | 95%CI | B | β | t | *P* | 95%CI | B | β | t | *P* | 95%CI |
| Food responsiveness | 0.258 | 0.287 | 3.311 | **0.001** | (0.104,0.413) | 0.308 | 0.338 | 3.440 | **0.001** | (0.130,0.486) | 0.285 | 0.312 | 3.184 | **0.002** | (0.107,0.462) |

CI: confidence interval.

^a^unadjusted model.

^b^adjusted model: adjusted for sex, age, birth weight, father's education level, mother's education level, parents' marital status and monthly household income.

^c^adjusted model: adjusted for sex, age, birth weight, father's education level, mother's education level, parents' marital status, monthly household income and positive item counts of oppositional defiant disorder, conduct disorder, and anxiety/depression.

**Table S4 Correlation of** **sleep habits with ADHD core symptoms and psychiatric comorbidities in children with ADHD and comorbid overweight or obesity.**

|  | 1 | 2 | 3 | 4 | 5 | 6 | 7 | 8 | 9 | 10 | 11 | 12 | 13 |
| --- | --- | --- | --- | --- | --- | --- | --- | --- | --- | --- | --- | --- | --- |
| 1 Attention deficit | - |  |  |  |  |  |  |  |  |  |  |  |  |
| 2 Hyperactivity/impulsivity | 0.230* | - |  |  |  |  |  |  |  |  |  |  |  |
| 3 Oppositional defiant disorder | 0.194* | 0.360** | - |  |  |  |  |  |  |  |  |  |  |
| 4 Conduct disorder | 0.225* | 0.349** | 0.519** | - |  |  |  |  |  |  |  |  |  |
| 5 Anxiety/depression | 0.049 | -0.045 | 0.260* | 0.163 | - |  |  |  |  |  |  |  |  |
| 6 Bedtime resistance | 0.024 | 0.036 | 0.018 | -0.102 | 0.091 | - |  |  |  |  |  |  |  |
| 7 Sleep onset delay | -0.108 | -0.021 | **-0.286*** | -0.042 | **-0.270*** | 0.137 | - |  |  |  |  |  |  |
| 8 Sleep duration | -0.126 | -0.137 | 0.008 | -0.135 | 0.068 | 0.201* | -0.028 | - |  |  |  |  |  |
| 9 Sleep anxiety | 0.007 | -0.019 | 0.112 | -0.046 | **0.179*** | 0.598** | -0.230* | 0.097 | - |  |  |  |  |
| 10 Night waking | 0.098 | -0.001 | -0.037 | 0.049 | 0.013 | 0.277** | 0.076 | 0.175 | 0.243** | - |  |  |  |
| 11 Parasomnias | 0.108 | 0.003 | 0.030 | 0.016 | **0.185*** | 0.300** | 0.002 | 0.209* | 0.357** | 0.742** | - |  |  |
| 12 Sleep disordered breathing | 0.134 | 0.094 | 0.047 | 0.137 | 0.038 | 0.205* | -0.053 | 0.046 | 0.301** | 0.656** | 0.805** | - |  |
| 13 Daytime sleepiness | 0.058 | -0.072 | 0.140 | 0.054 | **0.181*** | 0.059 | -0.114 | 0.214* | 0.228* | 0.459** | 0.505** | 0.397** | - |

* *P*<0.05, ** *P*<0.001

**Table S5 Kappa agreement statistics between two classification systems.**

| WHO BMIZ standard​ | Chinese standard | | Kappa |
| --- | --- | --- | --- |
|  | ADHD normal weight group (n=145) | ADHD overweight/obesity group (n=124) |  |
| ADHD normal weight group (n=136) | 135 | 1 | 0.918 |
| ADHD overweight/obesity group (n=133) | 10 | 123 |  |

**Table S6** **Demographic characteristics of the ADHD normal weight group and ADHD overweight/obesity group** **redefined using WHO BMIZ standard.**

|  | | WHO BMIZ standard | | | |
| --- | --- | --- | --- | --- | --- |
| Variable | | ADHD normal weight group (n=136) | ADHD overweight/obesity group (n=133) | t/X^2^ | *P* |
| BMI (kg/m^2^) | | 15.2 ± 1.6 | 23.1 ± 5.1 | -16.976 | **<0.001** |
| Sex | Male (%) | 102 (75.0%) | 108 (81.2%) | 1.511 | 0.219 |
| Age (years) | | 8.4 ± 1.3 | 8.7 ± 1.5 | -1.917 | 0.056 |
| ADHD subtypes | Inattention dominant type | 88 (64.7%) | 82 (61.7%) | 2.759 | 0.252 |
|  | Hyperactivity/Impulse dominant type | 15 (11.0%) | 9 (6.8%) |  |  |
|  | Combined type | 33 (24.3%) | 42 (31.6%) |  |  |
| Birth weight (kg) | | 3.4 ± 0.6 | 3.4 ± 0.6 | 0.016 | 0.988 |
| Birth weight | Low birth weight | 7 (5.1%) | 5 (3.8%) | 0.304 | 0.859 |
|  | Normal birth weight | 118 (86.8%) | 117 (88.0%) |  |  |
|  | Macrosomia | 11 (8.1%) | 11 (8.3%) |  |  |
| Gestational age (week) | | 39.1 ± 1.9 | 39.0 ± 2.2 | 0.576 | 0.565 |
| Gestational age | Preterm | 11 (8.1%) | 11 (8.3%) | 0.074 | 0.963 |
|  | Term | 119 (87.5%) | 117 (88.0%) |  |  |
|  | Post-term | 6 (4.4%) | 5 (3.8%) |  |  |
| Father's education level | Primary school and below | 6 (4.4%) | 10 (7.5%) | 1.535 | 0.464 |
|  | Junior/Senior School | 69 (50.7%) | 70 (52.6%) |  |  |
|  | Bachelor’s degree and above | 61 (44.9%) | 53 (39.8%) |  |  |
| Mother's education level | Primary school and below | 6 (4.4%) | 7 (5.3%) | 1.973 | 0.373 |
|  | Junior/Senior School | 57 (41.9%) | 66 (49.6%) |  |  |
|  | Bachelor’s degree and above | 73 (53.7%) | 60 (45.1%) |  |  |
| Parents' marital status | Married | 123 (90.4%) | 112 (84.2%) | 2.364 | 0.124 |
|  | Divorced/widowed/single | 13 (9.6%) | 21 (15.8%) |  |  |
| Residence | Urban area | 113 (83.1%) | 108 (81.2%) | 0.163 | 0.686 |
|  | Rural area | 23 (16.9%) | 25 (18.8%) |  |  |
| Monthly household income (¥) | ≤2000 | 5 (4.5%) | 7 (6.3%) | 6.448 | 0.168 |
|  | 2000-5000 | 39 (34.8%) | 31 (27.9%) |  |  |
|  | 5000-10000 | 39 (34.8%) | 52 (46.8%) |  |  |
|  | 10000-20000 | 21 (18.8%) | 11 (9.9%) |  |  |
|  | ≥20000 | 8 (7.1%) | 10 (9.0%) |  |  |
| Daily screen exposure time (hour) | <1 | 78 (59.1%) | 79 (61.2%) | 0.126 | 0.723 |
|  | ≥1 | 54 (40.9%) | 50 (38.8%) |  |  |
| Daily exercise time (hour) | <1 | 79 (59.4%) | 74 (57.4%) | 0.112 | 0.738 |
|  | ≥1 | 54 (40.6%) | 55 (42.6%) |  |  |

**Table S7 Eating behavior and sleep habit problems of the ADHD normal weight group and ADHD overweight/obesity group** **redefined using WHO BMIZ standard.**

|  |  | WHO BMIZ standard | | | |
| --- | --- | --- | --- | --- | --- |
|  |  | ADHD normal weight group (n=136) | ADHD overweight/obesity group (n=133) | t | *P* |
| Eating behavior problems | Satiety responsiveness | 10.6 ± 4.2 | 6.6 ± 3.9 | 7.936 | **<0.001** |
|  | Slowness in eating | 8.6 ± 4.2 | 5.1 ± 4.0 | 6.801 | **<0.001** |
|  | Fussiness | 10.6 ± 5.1 | 10.6 ± 4.5 | -0.022 | 0.982 |
|  | Emotional undereating | 6.1 ± 4.1 | 4.9 ± 3.8 | 2.480 | **0.014** |
|  | Food responsiveness | 4.3 ± 3.7 | 5.7 ± 4.5 | -2.688 | **0.008** |
|  | Enjoyment of food | 7.7 ± 4.3 | 10.1 ± 4.6 | -4.393 | **<0.001** |
|  | Desire to drink | 5.0 ± 3.4 | 5.0 ± 3.5 | -0.036 | 0.971 |
|  | Emotional overeating | 1.9 ± 2.6 | 1.7 ± 3.0 | 0.316 | 0.752 |
| Sleep habit problems | Bedtime resistance | 11.8 ± 2.0 | 11.8 ± 1.8 | 0.213 | 0.831 |
|  | Sleep onset delay | 2.6 ± 0.6 | 2.5 ± 0.7 | 0.599 | 0.550 |
|  | Sleep duration | 6.4 ± 1.4 | 6.5 ± 1.4 | -0.713 | 0.477 |
|  | Sleep anxiety | 7.0 ± 2.1 | 7.2 ± 2.2 | -0.851 | 0.396 |
|  | Night waking | 3.6 ± 1.2 | 4.1 ± 1.8 | -2.681 | **0.008** |
|  | Parasomnias | 8.6 ± 2.2 | 9.1 ± 3.5 | -1.407 | 0.159 |
|  | Sleep disordered breathing | 3.5 ± 1.2 | 3.9 ± 1.6 | -2.406 | **0.017** |
|  | Daytime sleepiness | 9.6 ± 2.5 | 10.3 ± 2.8 | -1.953 | 0.052 |
|  | Total sleep score | 48.9 ± 6.7 | 51.2 ± 10.0 | -2.175 | **0.031** |

**Table S8a Scores of attention deficit and hyperactivity/impulsivity, and** **psychiatric comorbidity status of the ADHD normal weight group and ADHD overweight/obesity group** **redefined using WHO BMIZ standard.**

|  | | WHO BMIZ standard | | | |
| --- | --- | --- | --- | --- | --- |
|  |  | ADHD normal weight group (n=136) | ADHD overweight/obesity group (n=133) | t | *P* |
| Attention deficit | | 19.6 ± 4.3 | 19.9 ± 4.1 | -0.442 | 0.659 |
| Hyperactivity/impulsivity | | 14.4 ± 6.2 | 14.7 ± 6.2 | -0.448 | 0.655 |
| Oppositional defiance | Absent | 80 (58.8%) | 84 (63.2%) | 0.531 | 0.466 |
|  | Present | 56 (41.2%) | 49 (36.8%) |  |  |
| Conduct disorder | Absent | 131 (96.3%) | 126 (94.7%) | 0.397 | 0.529 |
|  | Present | 5 (3.7%) | 7 (5.3%) |  |  |
| Anxiety/depression | Absent | 109 (80.1%) | 109 (82.0%) | 0.143 | 0.705 |
|  | Present | 27 (19.9%) | 24 (18.0%) |  |  |

**Table S8b Positive psychiatric comorbidity item counts of the ADHD normal weight group and ADHD overweight/obesity group redefined using WHO BMIZ standard.**

|  | ADHD normal weight group (n=136) | ADHD overweight/obesity group (n=133) | z | *P* |
| --- | --- | --- | --- | --- |
| Oppositional defiance disorder | 3.0 (1.0-5.0) | 2.0 (1.0-4.0) | -0.481 | 0.631 |
| Conduct disorder | 0.0 (0.0-1.0) | 0.0 (0.0-1.0) | -0.044 | 0.965 |
| Anxiety/depression | 1.0 (0.0-2.0) | 0.0 (0.0-2.0) | -0.967 | 0.333 |

**Table S9 Associations of eating behavior and sleep habit problems on the presence or absence of WHO BMIZ defined overweight or obesity in children with ADHD.**

|  | WHO BMIZ standard | | | | | | | | | | | | | | |
| --- | --- | --- | --- | --- | --- | --- | --- | --- | --- | --- | --- | --- | --- | --- | --- |
|  | Model 1^a^ | | | | | Model 2^b^ | | | | | Model 3^c^ | | | | |
|  | B | Wald | *P* | OR | 95%CI | B | Wald | *P* | OR | 95%CI | B | Wald | *P* | OR | 95%CI |
| Food responsiveness | 0.061 | 2.551 | 0.110 | 1.063 | (0.986, 1.146) | 0.063 | 2.525 | 0.112 | 1.065 | (0.985, 1.150) | 0.063 | 2.515 | 0.113 | 1.065 | (0.985, 1.152) |
| Enjoyment of food | 0.021 | 0.332 | 0.565 | 1.022 | (0.950, 1.099) | 0.012 | 0.098 | 0.754 | 1.012 | (0.939, 1.090) | 0.014 | 0.132 | 0.717 | 1.014 | (0.941, 1.093) |
| Satiety responsiveness | -0.157 | 14.028 | **<0.001** | 0.854 | (0.787, 0.928) | -0.161 | 13.862 | **<0.001** | 0.851 | (0.782, 0.926) | -0.163 | 13.960 | **<0.001** | 0.849 | (0.780, 0.925) |
| Slowness in eating | -0.112 | 9.547 | **0.002** | 0.894 | (0.832, 0.960) | -0.117 | 9.560 | **0.002** | 0.889 | (0.826, 0.958) | -0.116 | 9.340 | **0.002** | 0.890 | (0.827, 0.959) |
| Emotional undereating | -0.051 | 1.640 | 0.200 | 0.950 | (0.879, 1.027) | -0.046 | 1.263 | 0.261 | 0.955 | (0.882, 1.035) | -0.052 | 1.491 | 0.222 | 0.950 | (0.874, 1.032) |
| Night waking | 0.140 | 1.649 | 0.199 | 1.151 | (0.929, 1.426) | 0.148 | 1.729 | 0.189 | 1.160 | (0.930, 1.446) | 0.152 | 1.753 | 0.185 | 1.164 | (0.930, 1.456) |
| Sleep disordered breathing | 0.094 | 0.620 | 0.431 | 1.098 | (0.869, 1.388) | 0.096 | 0.623 | 0.430 | 1.101 | (0.868, 1.396) | 0.082 | 0.443 | 0.506 | 1.085 | (0.853, 1.379) |
| Daytime sleepiness | 0.043 | 0.708 | 0.400 | 1.044 | (0.944, 1.154) | 0.059 | 1.213 | 0.271 | 1.061 | (0.955, 1.179) | 0.074 | 1.789 | 0.181 | 1.077 | (0.966, 1.200) |

CI: confidence interval.

^a^unadjusted model.

^b^adjusted model: adjusted for sex, age, and daily exercise time.

^c^adjusted model: adjusted for sex, age, daily exercise time, and positive item counts of oppositional defiant disorder, conduct disorder, and anxiety/depression.

**Table S10 Correlation between sleep habits and BMI in children with ADHD and comorbid** **WHO BMIZ defined overweight or obesity.**

|  | WHO BMIZ standard | | | | | | | | | | | | | | |
| --- | --- | --- | --- | --- | --- | --- | --- | --- | --- | --- | --- | --- | --- | --- | --- |
|  | Model1^a^ | | | | | Model2^b^ | | | | | Model3^c^ | | | | |
|  | B | β | t | P | 95%CI | B | β | t | *P* | 95%CI | B | β | t | *P* | 95%CI |
| Sleep duration | -0.744 | -0.204 | -2.385 | **0.018** | (-1.362, -0.127) | -0.775 | -0.213 | -2.436 | **0.016** | (-1.405, -0.145) | -0.776 | -0.213 | -2.369 | **0.019** | (-1.424, -0.127) |

CI: confidence interval.

^a^unadjusted model.

^b^adjusted model: adjusted for sex, ADHD subtypes, daily screen exposure time and daily exercise time.

^c^adjusted model: adjusted for sex, ADHD subtypes, daily screen exposure time, daily exercise time and positive item counts of oppositional defiant disorder, conduct disorder, and anxiety/depression.

**Table S11 Correlation of food responsiveness with attention deficit in children with ADHD and comorbid WHO BMIZ defined overweight or obesity.**

|  | WHO BMIZ standard | | | | | | | | | | | | | | |
| --- | --- | --- | --- | --- | --- | --- | --- | --- | --- | --- | --- | --- | --- | --- | --- |
|  | Model1^a^ | | | | | Model2^b^ | | | | | Model3^c^ | | | | |
|  | B | β | t | *P* | 95%CI | B | β | t | *P* | 95%CI | B | β | t | *P* | 95%CI |
| Food responsiveness | 0.223 | 0.247 | 2.923 | **0.004** | (0.072, 0.373) | 0.245 | 0.270 | 2.821 | **0.006** | (0.073, 0.418) | 0.222 | 0.244 | 2.525 | **0.013** | (0.047, 0.396) |

CI: confidence interval.

^a^unadjusted model.

^b^adjusted model: adjusted for sex, age, birth weight, father's education level, mother's education level, parents' marital status and monthly household income.

^c^adjusted model: adjusted for sex, age, birth weight, father's education level, mother's education level, parents' marital status, monthly household income and positive item counts of oppositional defiant disorder, conduct disorder, and anxiety/depression.
